# Clinical and Microbiological Characteristics of Perianal Infections in Adult Patients with Acute Leukemia

**DOI:** 10.1371/journal.pone.0060624

**Published:** 2013-04-05

**Authors:** Chien-Yuan Chen, Aristine Cheng, Shang-Yi Huang, Wang-Huei Sheng, Jia-Hau Liu, Bo-Sheng Ko, Ming Yao, Wen-Chien Chou, Hui-Chi Lin, Yee-Chun Chen, Woei Tsay, Jih-Luh Tang, Shan-Chwen Chang, Hwei-Fang Tien

**Affiliations:** 1 Department of Internal Medicine, National Taiwan University Hospital, Taipei, Taiwan; 2 Division of Infectious Disease, Department of Internal Medicine, Far-East Memorial Hospital, New Taipei City, Taiwan; 3 Department of Laboratory Medicine, National Taiwan University Hospital, Taipei, Taiwan; 4 Infection Control Center, National Taiwan University Hospital, Taipei, Taiwan; 5 Tai-Cheng Stem Cell Therapy Center, National Taiwan University Hospital, Taipei, Taiwan; Charité, Campus Benjamin Franklin, Germany

## Abstract

**Background:**

Perianal infection is a common problem for patients with acute leukemia. However, neutropenia and bleeding tendency are relatively contraindicated to surgical intervention. The epidemiology, microbiology, clinical manifestations and outcomes of perianal infection in leukemic patients are also rarely discussed.

**Method:**

The medical records of 1102 adult patients with acute leukemia at a tertiary medical center in Taiwan between 2001 and 2010 were retrospectively reviewed and analyzed.

**Result:**

The prevalence of perianal infection was 6.7% (74 of 1102) in adult patients with acute leukemia. Twenty-three (31%) of the 74 patients had recurrent episodes of perianal infections. Patients with acute myeloid leukemia had higher recurrent rates than acute lymphoblastic leukemia patients (p = 0.028). More than half (*n* = 61, 53%) of the perianal infections were caused by gram-negative bacilli, followed by gram-positive cocci (*n* = 36, 31%), anaerobes (*n* = 18, 15%) and *Candida* (*n* = 1, 1%) from pus culture. Eighteen patients experienced bacteremia (*n* = 24) or candidemia (*n* = 1). Overall 41 (68%) of 60 patients had polymicrobial infection. *Escherichia coli* (25%) was the most common micro-organism isolated, followed by *Enterococcus* species (22%), *Klebsiella pneumoniae* (13%), and *Bacteroides* species (11%). Twenty-five (34%) of 74 patients received surgical intervention. Acute leukemia patients with surgically managed anal fistulas tended to have fewer recurrences (p = 0.067). Four (5*%) patients died within 30 days after diagnosis of perianal infection. Univariate analysis of 30-day survival revealed the elderly (≧ 65 years) (p = 0.015) and patients with shock (p<0.001) had worse outcome. Multivariate analysis showed septic shock to be the* independent predictive factor of 30-day crude mortality of perianal infections (p = 0.016).

**Conclusion:**

Perianal infections were common and had high recurrence rate in adult patients with acute leukemia. Empirical broad-spectrum antibiotics with anaerobic coverage should be considered. Shock independently predicted 30-day crude mortality. Surgical intervention for perianal infection remains challenging in patients with acute leukemia.

## Introduction

Perianal abscess and anal fistulas are the acute and chronic manifestations of perianal infection [1]–[3]. It is estimated one-third of patients with perianal abscess may be complicate by chronic anal fistula formation [2], [3]. Perianal infections are often accompanied by severe pain, swelling, constipation, and may cause systemic infection and life-threatening sepsis [4], [5].

Infection is one of the most serious complications in patients with acute leukemia receiving chemotherapy [6], [7]. Perianal infection is not uncommon in patients with hematological malignancy [8]–[14]. Leukemic patients receiving chemotherapy and radiotherapy with resultant anal mucosa damage can provide an essential port of entry for pathogens during nadir stage [8]–[14]. The Disease spectrum of perianal infection might range from mild local cellulitis to life-threatening sepsis in patients with chemotherapy related neutropenia. Furthermore, neutropenic patients are unable to mount an adequate inflammatory response and are susceptible to infection by less virulent micro-organisms, consequently they may have unusual presentations of infection [15]. Not only can perianal infection cause mortality, but anal pain and discomfort also significantly impact the quality of life in patients with acute leukemia [14].

The principal management of perianal abscess and fistulas is surgery in the general population [1], [2]. Since leukemic patients are usually neutropenic with bleeding tendencies, surgical intervention could cause sepsis and poor wound healing [13]. Therefore, the optimal time for surgical treatment is still questionable in leukemic patients with perianal infection. To understand the prevalence, disease manifestations and outcomes of perianal infection in modern care, we retrospectively reviewed the medical records of 1102 acute leukemic patients in the National Taiwan University Hospital (NTUH) between 2001 and 2010 and analyze the clinical characteristic, microbiology and outcomes of perianal infection in acute leukemic adult patients.

## Patients and Methods

### Ethics Statement

The Institutional Review Board National Taiwan University Hospital Research Ethics Committee waived the need for written informed consent from the participants in the retrospective review of medical record and approved this study. This research conformed to the Helsinki Declaration and local legislation, and was approved by the Institutional Review Board National Taiwan University Hospital Research Ethics Committee.

### Hospital setting and patients

National Taiwan University Hospital (NTUH) is a 2500-bed teaching hospital in northern Taiwan that provides both primary and tertiary care. We retrospectively analyzed the clinical manifestation, laboratory and microbiological data, and outcome of adult patients during January 2001 to December 2010. The overall 30-day crude mortality of these patients were assessed.

### Definitions

Perianal infections included perianal abscess and fistula infection. Perianal abscess was defined as the presence of an erythematous firm or fluctuant tender mass located around the anus. Anal fistula was defined as an opening in the skin near the anus that leads into a blind pouch or may connect through a tunnel with the rectal canal. Sepsis was diagnosed in patients with signs of systemic inflammatory response syndrome (including two or more of the following criteria: temperature greater than 38°C or less than 36°C; heart rate greater than 90 beats/min; respiratory rate greater than 20 breaths/min or pCO2 less than 32 torr; or white blood cell count greater 12000 cells/mm^3^, less than 4000 cells/mm^3^, or higher than 10% band forms) and clinically suspected infection. Mortality attributable to perianal infection was defined as death within 30 days after diagnosis of perianal infection while the infection still active and no other possible cause of death was found.

### Statistical analysis

Categorical variables were compared using the chi-squared test. The significance level was set at 0.05 and all *p* values were two-tailed. Univariate analysis of 30 day survival was performed by Kaplan-Meier method and log rank test. Multivariate analyses with Cox regression analysis were performed. All statistical analyses were performed using the statistical package SPSS for Windows (Version 18, SPSS, and Chicago, IL, USA).

## Results

### Epidemiology and clinical characteristics

During the study period, a total of 74 (6.7%) of 1102 adult patients with acute leukemia, included 59 (6.9%) of 853 patients with acute myeloid leukemia (AML) and 15 (6.0%) of 249 patients with acute lymphoblastic leukemia (ALL) were diagnosed with perianal infections. There were 57 men and 17 women with a median age of 43 years (range 18–77 years). The clinical characteristics were shown in Table 1. Sixty-six patients had perianal abscess, 30 patients had anal fistula infection, and 22 patients had both perianal abscess and fistula. Perianal infections were the initial presentation of acute leukemia in 14 of 74 patients (19%). Elderly patients (age ≧65 years) had less recurrent perianal infections than those with age <65 years (p = 0.05). AML patients had higher rates of recurrent perianal infections than ALL patients (p = 0.028).

**Table 1:** Clinical characteristics of 74 adult leukemic patients with perianal abscess and/or fistula infection.

Clinical characters	Patient number		P Value
	Recurrent n = 23(%)	Non-recurrent n = 51(%)	
Elder (Age> = 65 year)	0(0)	9(18)	0.050
Gender(male)	18(78)	39(76)	>0.999
Diabetes mellitus	0(0)	2(4)	>0.999
Perianal abscess	21(91)	45(88)	>0.999
Anal fistula	8(35)	29(57)	0.612
Acute myeloid leukemia	22(96)	37(73)	0.028
Remission disease status	10(43)	14(27)	0.190
Chemotherapy	22(96)	44(86)	0.422
Neutropenia	21(91)	44(86)	0.711
Transplantation	0(0)	3(6)	0.548
Shock	0(0)	6(12)	0.097
Surgery	7(30)	18(35)	0.793

### Microbiology

The pathogens isolated from pus cultures and blood cultures were shown in Table 2. Overall, 116 and 25 microorganisms were isolated from pus and blood, respectively, from sixty patients. Forty-one (68%) of 60 patients had polymicrobial infections (2 to 5 pathogens). Gram-negative bacilli were the most predominant isolates (53% of pus culture isolates and 63% of blood culture isolates), followed by Gram-positive cocci, anaerobes and Candida (31%, 15% and 1% of pus culture isolates, and 25%, 8% and 1% of blood culture isolates, respectively). *E. coli* (25%) was the most common isolate, followed by *Enterococcus* species (22%), *Klebsiella pneumoniae* (13%) and *Bacteroides* species (11%). Three (12%) of 25 *Enterococcus* species isolated possessed vancomycin resistance, and 3 (10%) of 29 *E. coli* produced extended spectrum beta-lactamases (ESBL) in the pus culture.

**Table 2:** Microbiology of 74 adult leukemic patients with perianal abscess and fistula infection.

Causative pathogen	Overall n = 130(%)	Pus culture n = 116 (%)	Blood culture n = 25 (%)
Gram positive cocci	42(32)	36 (31)	6 (24)
Enterococcus spp.	28(22)	25 (22)	3(12)
Coagulase-negative Staphylococcus	5(4)	4(3)	1(4)
Streptococcus viridans	4(3)	4(3)	0(0)
Staphylococcus epidermidis	4(3)	2(2)	2(8)
Staphylococcus aureus	1(1)	1(1)	0(0)
Gram negative bacilli	67(52)	61 (53)	15 (60)
Escherichia coli	30(23)	29 (25)	6(24)
Klebsiella pneumoniae	16(12)	15 (13)	1(4)
Proteus mirabilis	9(7)	9 (8)	1(4)
Enterobacter cloacae	5(4)	4(3)	2(8)
Aeromonas hydrophilia	3(2)	2(2)	2(8)
Pseudomonas aeruginosa	2(2)	1(1)	2(8)
Citrobacter frenudii	1(1)	1(1)	0(0)
Comamonas testosteroni	1(1)	0(0)	1(4)
Moraxella species	1(1)	0(0)	1(4)
Anaerobic pathogens	21(16)	18(15)	2(8)
Bacteroides fragilis	13(10)	13(11)	1(4)
Peptostreptococcus	2(2)	2(2)	0(0)
Clostridium species	2(2)	1(1)	1(4)
Prevotella species	1(1)	1(1)	0(0)
Veillonella species	1(1)	1(1)	0(0)
Candida	2(2)	1(1)	2(8)
Candida tropicalis	1(1)	1(1)	1(4)
Candida albicans	1(1)	0(0)	1(4)

Nine patients with 10 pathogens isolated in both blood and pus culture, including E.coli 4, Pseudomonas aeruginosa 1, Proteus mirabilis 1, Enterococcus specie 1, Bacteroides specie 1, Aeromonas hydrophila and Enterobacter cloacae 1.

### Treatment and outcomes

The prescribed antimicrobials for perianal abscess and anal fistula were heterogeneous, including 17 patients treated with carbapenem alone, 9 patients treated with carbapenem and vancomycin, 2 patients treated with carbapenem and piperacillin/tazobactam, 10 patients treated with cefepime alone, 9 patients treated with cefepime and metronidazole, 5 patients treated with cefepime and vancomycin; 9 patients treated with piperacillin/tazobactam alone; 3 patients treated with ticarcillin and clavulanate and aminoglycoside, one patient treated with ticarcillin/clavulanate and vancomycin, three patients treated with amoxicillin/clavulanate and aminoglycoside, and 5 patients treated with second generation or third generation cephalosporin and aminoglycoside. Twelve patients received a concomitant antifungal agent; 10 patients received fluconazole and 2 patients, amphotericin B. Whether the empirical antibiotics appropriately covered the isolated pathogens within 48 hour or not (early discordant antimicrobial therapy, 6.8% vs. 5.9%, p = 0.917) and coverage of anaerobic pathogens by either the initial or definitive antibiotics did not influence the 30 day outcome (8.1% vs. 0%, p = 0.315).

Twenty-five (34%) of 74 patients had undergone surgical intervention. Nineteen patients received incision and drainage, three patients received fistulectomy, two patients received fistulotomy, and one patient whose perianal abscess was complicated with Fournier's gangrene received debridement and fasciotomy. Four patients received two or three times incision and drainage. Five (20%) patients developed complications after surgical intervention, including four patients with sepsis after surgical intervention and one patient with poor wound healing. Nineteen (29%) of 65 neutropenic patients and 6 (67%) of 9 non-neutropenic patients received surgical intervention, neutropenic patients were more likely to not receive surgical intervention than non-neutropenic patients (p = 0.054). Surgical intervention for acute leukemic patients with anal fistulas showed a trend towards fewer recurrence (p = 0.067), but surgical intervention did not appear to decrease recurrences of perianal abscesses. The clinical characteristics of patients receiving surgery compared to those managed conservatively were no different in terms of age, gender, leukemia type, transplantation, and so on. Only three patients had magnetic resonance imaging to evaluate the anal lesion, including the patient with Fournier's gangrene. He had magnetic resonance imaging to evaluate the extent of lesion before surgery. Two other patients had ano-vaginal fistula. These two patients had relapsed or refractory leukemia, so they did not receive surgical intervention.

Univariate analysis of 30-day survival was performed with the Kaplan-Meier method and log-rank test (Table 3). Elderly (p = 0.015) and patients with shock status (p<0.001) had worse outcome. Multivariate analysis of 30-day outcome was performed with Cox regression analysis. Shock is the only independent factor predictive of 30-day crude mortality in hematologic patients with perianal infections (p = 0.016).

**Table 3:** Univariate and multivariate analysis of 30-day outcome of 74 patients with perianal abscess and anal fistula infection.

Clinical characters	Survival	Died	univariate	multivariate
	n = 70(%)	n = 4(%)	P value	P value
Elderly (Age > = 65 years)	7(10)	2(50)	0.015	NS
Gender(Male)	54(77)	3(75)	0.901	NS
Perianal infection as initial presentation	13(19)	1(25)	0.758	NS
Perianal abscess	62(89)	4(100)	0.481	NS
Anal fistula	29(41)	1(25)	0.514	NS
Acute myeloid leukemia	56(80)	3(75)	0.832	NS
Remission disease status	24(34)	0(0)	0.159	NS
Chemotherapy	63(90)	3(75)	0.317	NS
Neutropenia	61(87)	4(100)	0.451	NS
Transplantation	3(4)	0(0)	0.678	NS
Surgical intervention	24(34)	1(25)	0.700	NS
Shock	3(4)	3(75)	<0.001	0.016

## Discussion

Although the supportive management of patients has considerably improved in the past several decades, the prevalence of perianal infections in patients with acute leukemia of our study (6.7%) is similar to the result of previous studies (5∼9%) [8], [9], [15]. Even this disease entity is not common, perianal abscess and anal fistulas have long existed as problems for patients with acute leukemia, impacting the morbidity, mortality, and quality of life of these vulnerable patients.

The type of leukemia did not appreciably alter the prevalence of perianal abscess and/or anal fistula. Perianal infections usually recur in the period of neutropenia after chemotherapy. Patients with acute myeloid leukemia generally receive high dose cytarabine based chemotherapy with significantly worse neutropenia and mucositis than patients with acute lymphoblastic leukemia. This may explain why patients with acute myeloid leukemia had higher recurrence rate of perianal infection than patients with acute lymphoblastic leukemia (p = 0.028). Patients who were aged above 65 years had a trend towards lower recurrence rate than the younger patients. This lower recurrence rate could be an artifact related to the shorter overall survival of elderly patients. A larger scale study may be needed to confirm the findings of our relatively small single center study.

Few investigations have been held on the microbiology of perianal abscess and anal fistula infection in acute leukemia patients [8], [9]. Vanhueverzwyn et al. and Barnes and colleagues found *Pseudomonas aeruginosa* and *E. coli* were the predominant pathogens [8], [9]. In contrast, we found *E. coli, Enterococcus* and *Klebsiella pneumoniae* were the leading pathogens. *Pseudomonas aeruginosa* comprised only two percent of the cultured isolates. Although febrile neutropenia caused by *Bacteroides* species were few (about 3%) [7], [16], *Bacteroides* species represented 10% overall of the isolated pathogens in this study. Multivariate analysis revealed shock as the independent factor associated with poor outcomes for these leukemic patients with perianal infection. The antimicrobial coverage of anaerobes could be better control of perianal infection in hematological patients. Liu et al and Eykyn et al. reported that *E. coli* and *Bacteroides* species were the leading pathogens in the general population [17], [18]. Enterococcus species occupied 22% of all pathogens isolated in the study. Leukemia patients are frequently given broad spectrum antibiotics for febrile neutropenia. Gram negative bacteria decreased under broad spectrum antibiotics whilst gram positive enterococcus increased. Tuberculosis and actinomycosis have also been reported to cause perianal infections among neutropenic patients [19], [20], but there were no cases found in this study. Our results reasonable suggest that Enterobacteriaceae are the most common causative pathogens of perianal infection, not streptococci or staphylocci, which are the most common causative pathogens of other site of skin and soft tissue infections.

The principal management of perianal abscess and anal fistula is surgery [1], [2]. Abscess should be drained in a timely manner. Delayed or inadequate treatment may cause extensive life-threatening infection and sepsis [1]. In this study, neither early discordant antimicrobial therapy nor inadequate antimicrobial coverage of anaerobic pathogens influence the 30-day mortality. The findings might reflect low mortality in this disease entity and effect of spontaneous pustular drainage or surgical debridement for better infection control. Meta-analysis by Malik and colleagues showed fistula surgery with abscess drainage significantly reduces recurrence or persistence of abscess fistula in general population [21]. The recurrence rate is 3 to 5% in the general population [17], [22]. The recurrence of perianal abscess and anal fistula infection is 31% in this study. This high recurrence rate underscores the importance of perianal disease for patients with acute leukemia. However, patients with acute leukemia commonly develop neutropenia and thrombocytopenia after chemotherapy. Moreover wound healing is impaired and sepsis may worsen after surgical intervention. The optimal time for surgical treatment remains a challenge for leukemic patients with perianal abscess and fistulas. Clinically, a surgeon may act conservatively if the patients' leukemia is not in remission. Some novel treatment such as plug in anal fistula may be an option for such leukemic patients [23], [24]. Recently, MRI exquisitely depicts the perianal anatomy and shows the fistulous tracks and their associated ramifications and abscesses [25], [26]. MRI appears to be a good tool to evaluate leukemic patients with perianal lesions, especially in the patients with complex anal fistula and complicated abscess.

There are some limitations to this study. First, the disease spectrum of perianal infection might range from mild local heat and tenderness to life threatening sepsis, especially in patients with hematologic malignancies received chemotherapy. In order to clearly define this issue, we limit the disease category specific to perianal abscess or anal fistula, which are more severe clinical condition of perianal infection. Therefore, the actual prevalence of perianal infection could be underestimated. Second, isolation of micro-organism from purulent discharge from an unsterile site is not perfect for identification of causative pathogens. In this retrospective study, we found that the pathogens derived from blood and discharge is similar (table 2). Therefore, we suggest the result of microbiology could represent partly the real pathogens of ano-rectal infection. Third, the detailed anatomic classifications of anal fistulas could not be obtained and there is no data to detail their miserable quality of life because of the retrospective study design.

### Conclusion

Perianal abscesses and anal fistulas are relatively common complications among adult patients with acute leukemia. Broad-spectrum antibiotics with anaerobic coverage are indicated for polymicrobial infection. Shock is the independent poor prognostic factor in adult leukemic patients with perianal infection.
